# Supplementary material for: An examination of early socioeconomic status and neighborhood disadvantage as independent predictors of antisocial behavior: A longitudinal adoption study
Source: PLoS One. 2024 Apr 29;19(4):e0301765. doi: 10.1371/journal.pone.0301765 (PMC11057761; doi:10.1371/journal.pone.0301765)
Supplement: S1 Table — (DOCX) [file pone.0301765.s001.docx]

Table S1. Frequencies for Parent and Teacher Reported Externalizing Scales

| Age | Parent Report | | | Teacher Report | | |
| --- | --- | --- | --- | --- | --- | --- |
|  | N | Category | Frequency | N | Category | Frequency |
| 4 | 603 | 0 | 114 | N/A |  |  |
|  |  | 1 | 138 |  |  |  |
|  |  | 2 | 189 |  |  |  |
|  |  | 3 | 162 |  |  |  |
| 7 | 583 | 0 | 146 | 544 | 0 | 159 |
|  |  | 1 | 156 |  | 1 | 151 |
|  |  | 2 | 154 |  | 2 | 136 |
|  |  | 3 | 127 |  | 3 | 98 |
| 8 | N/A |  |  | 498 | 0 | 140 |
|  |  |  |  |  | 1 | 153 |
|  |  |  |  |  | 2 | 112 |
|  |  |  |  |  | 3 | 93 |
| 9 | 569 | 0 | 186 | 495 | 0 | 129 |
|  |  | 1 | 143 |  | 1 | 140 |
|  |  | 2 | 131 |  | 2 | 129 |
|  |  | 3 | 109 |  | 3 | 97 |
| 10 | 575 | 0 | 199 | 467 | 0 | 138 |
|  |  | 1 | 142 |  | 1 | 100 |
|  |  | 2 | 117 |  | 2 | 120 |
|  |  | 3 | 117 |  | 3 | 109 |
| 11 | 511 | 0 | 187 | 431 | 0 | 136 |
|  |  | 1 | 128 |  | 1 | 119 |
|  |  | 2 | 103 |  | 2 | 98 |
|  |  | 3 | 93 |  | 3 | 78 |
| 12 | 553 | 0 | 210 | 397 | 0 | 140 |
|  |  | 1 | 129 |  | 1 | 100 |
|  |  | 2 | 110 |  | 2 | 87 |
|  |  | 3 | 104 |  | 3 | 70 |
| 13 | 494 | 0 | 190 | 361 | 0 | 134 |
|  |  | 1 | 120 |  | 1 | 94 |
|  |  | 2 | 86 |  | 2 | 70 |
|  |  | 3 | 98 |  | 3 | 63 |
| 14 | 508 | 0 | 224 | 308 | 0 | 127 |
|  |  | 1 | 114 |  | 1 | 85 |
|  |  | 2 | 82 |  | 2 | 53 |
|  |  | 3 | 88 |  | 3 | 43 |
| 15 | 376 | 0 | 172 | 269 | 0 | 119 |
|  |  | 1 | 81 |  | 1 | 73 |
|  |  | 2 | 56 |  | 2 | 45 |
|  |  | 3 | 67 |  | 3 | 32 |
| 16 | 570 | 0 | 231 | N/A |  |  |
|  |  | 1 | 117 |  |  |  |
|  |  | 2 | 102 |  |  |  |
|  |  | 3 | 120 |  |  |  |

Note: Categories are as follows:

0 = “not true”, 1 = “somewhat or sometimes true”, 2 = “very true or often true”.
