# Supplementary material for: An examination of early socioeconomic status and neighborhood disadvantage as independent predictors of antisocial behavior: A longitudinal adoption study
Source: PLoS One. 2024 Apr 29;19(4):e0301765. doi: 10.1371/journal.pone.0301765 (PMC11057761; doi:10.1371/journal.pone.0301765)
Supplement: S2 Table — (DOCX) [file pone.0301765.s002.docx]

Table S2. Descriptive Statistics for Parent and Teacher Reported Aggression and Delinquency Scales

| **Age** | Parent Report | | | | | Teacher Report | | | | |
| --- | --- | --- | --- | --- | --- | --- | --- | --- | --- | --- |
|  | Delinquency | | | Aggression | | Delinquency | | | Aggression | |
|  | N | Mean | SD | Mean | SD | N | Mean | SD | Mean | SD |
| **4** | 550 | 1.42 | 1.34 | 7.82 | 5.28 | N/A |  |  |  |  |
| **7** | 547 | 1.39 | 1.42 | 6.62 | 4.92 | 513 | 0.85 | 1.43 | 5.09 | 7.39 |
| **8** | N/A |  |  |  |  | 471 | 0.75 | 1.38 | 4.94 | 6.95 |
| **9** | 534 | 1.21 | 1.48 | 6.26 | 5.34 | 469 | 0.76 | 1.42 | 5.13 | 7.01 |
| **10** | 545 | 1.21 | 1.54 | 6.13 | 5.23 | 446 | 0.83 | 1.41 | 5.82 | 7.56 |
| **11** | 485 | 1.15 | 1.63 | 5.86 | 5.20 | 419 | 0.79 | 1.36 | 4.71 | 6.61 |
| **12** | 533 | 1.19 | 1.62 | 5.95 | 5.31 | 387 | 0.79 | 1.43 | 4.21 | 6.18 |
| **13** | 453 | 1.30 | 1.96 | 5.67 | 5.40 | 336 | 0.81 | 1.59 | 4.21 | 6.60 |
| **14** | 499 | 1.37 | 2.08 | 5.16 | 5.29 | 302 | 0.87 | 1.80 | 3.34 | 6.22 |
| **15** | 358 | 1.65 | 2.56 | 4.92 | 5.29 | 256 | 0.88 | 1.66 | 2.82 | 5.21 |
| **16** | 570 | 2.09 | 3.10 | 5.55 | 5.53 | N/A |  |  |  |  |
