# Supplementary material for: An examination of early socioeconomic status and neighborhood disadvantage as independent predictors of antisocial behavior: A longitudinal adoption study
Source: PLoS One. 2024 Apr 29;19(4):e0301765. doi: 10.1371/journal.pone.0301765 (PMC11057761; doi:10.1371/journal.pone.0301765)
Supplement: S3 Table — (DOCX) [file pone.0301765.s003.docx]

Table S3. Correlations Between SES Variables and ND in Adoptees

|  | 1 | 2 | 3 | 4 | 5 | 6 | 7 |
| --- | --- | --- | --- | --- | --- | --- | --- |
| 1. Biological GF's Highest Completed Grade | 1 | .60*** | .47*** | .03 | .01 | -.03 | -.12 |
| 2. Biological GM's Highest Completed Grade | .60*** | 1 | .38*** | -.10 | .04 | -.11 | -.03 |
| 3. Biological GF's NORC | .53*** | .44*** | 1 | .02 | .04 | .10 | .00 |
| 4. Adoptive Father's Highest Completed Grade | .11 | .18^X^ | .05 | 1 | .51*** | .59*** | .05 |
| 5. Adoptive Mother's Highest Completed Grade | .12 | .11 | .08 | .49*** | 1 | .33*** | .09 |
| 6. Adoptive Father's NORC Score | .07 | .17* | -.01 | .65*** | .33*** | 1 | .06 |
| 7. Adoptive Parent’s ND | .00 | -.13 | -.23* | -.01 | .12 | -.05 | 1 |

^X^*p = .*03, **p* = .04, ****p <* .001

Note: Adopted girls above diagonal, adopted boys below diagonal. “GF” = grandfather, “GM” = grandmother.
