# Supplementary material for: An examination of early socioeconomic status and neighborhood disadvantage as independent predictors of antisocial behavior: A longitudinal adoption study
Source: PLoS One. 2024 Apr 29;19(4):e0301765. doi: 10.1371/journal.pone.0301765 (PMC11057761; doi:10.1371/journal.pone.0301765)
Supplement: S4 Table — (DOCX) [file pone.0301765.s004.docx]

Table S4. Correlations Between SES Variables and ND in Nonadoptees

|  | 1 | 2 | 3 | 4 |
| --- | --- | --- | --- | --- |
| 1. Biological Father's Highest Completed Grade | 1 | .50*** | .55*** | -.15 |
| 2. Biological Mother's Highest Completed Grade | .33*** | 1 | .35*** | -.09 |
| 3. Biological Father's NORC Score | .35*** | .10 | 1 | -.09 |
| 4. Biological Parent’s ND | -.12 | -.05 | .08 | 1 |

****p <* .001

Note: Nonadopted girls above diagonal, nonadopted boys below diagonal.
