# Supplementary material for: An examination of early socioeconomic status and neighborhood disadvantage as independent predictors of antisocial behavior: A longitudinal adoption study
Source: PLoS One. 2024 Apr 29;19(4):e0301765. doi: 10.1371/journal.pone.0301765 (PMC11057761; doi:10.1371/journal.pone.0301765)
Supplement: S5 Table — (DOCX) [file pone.0301765.s005.docx]

Table S5. Parent Reported ASB Slope, Intercept, and Correlations Between Slope and Intercept

|  | Slope | | Intercept | | Slope with Intercept |
| --- | --- | --- | --- | --- | --- |
|  | Mean | Variance | Mean | Variance |  |
| Adoptees |  |  |  |  |  |
| Girls | -0.29* | 1.01* | 0.00 | 0.60*** | -0.01 |
| Boys | -0.45** | 0.72** | 0.25 | 0.90*** | -0.01 |
| Nonadoptees |  |  |  |  |  |
| Boys | -1.13*** | 0.71** | -0.19 | 0.52*** | 0.01 |
| Girls | -.94*** | 0.87*** | 0.25 | 0.67*** | -0.18 |

**p <*.05, ***p* < .01, ****p* ≤ .001

*Note:* Standardized means and correlations between slope and intercept reported.
