# Supplementary material for: An examination of early socioeconomic status and neighborhood disadvantage as independent predictors of antisocial behavior: A longitudinal adoption study
Source: PLoS One. 2024 Apr 29;19(4):e0301765. doi: 10.1371/journal.pone.0301765 (PMC11057761; doi:10.1371/journal.pone.0301765)
Supplement: S6 Table — (DOCX) [file pone.0301765.s006.docx]

Table S6. Correlations Between Parent Reported ASB Intercept and Slope, ND, and Biological and Adoptive Parent SES in Adoptees

|  | Intercept | | Slope | |
| --- | --- | --- | --- | --- |
| *N =* 415 | Adoptive Parent SES | | | |
|  | *r* | *p* | *r* | *p* |
| Girls | -.05 [-.26, .17] | .67 | -.02 [-.25, .21] | .87 |
| Boys | -.19 [-.39, .01] | .06 | .11 [-.17, .39] | .43 |
| *N =* 357 | Biological Parent SES | | | |
|  | *r* | *p* | *r* | *p* |
| Girls | .13 [-.12, .37] | .30 | .20 [-.07, .47] | .14 |
| Boys | .03 [-.17, .22] | .77 | -.09 [-.39, .20] | .54 |
| *N =* 374 | ND | | | |
|  | *r* | *p* | *r* | *p* |
| Girls | .03 [-.22, .28] | .80 | -.18 [-.45, .09] | .18 |
| Boys | .02 [-.22, .26] | .85 | .22 [-.18, .62] | .29 |

*Note:* Standardized correlations reported.
