# Supplementary material for: An examination of early socioeconomic status and neighborhood disadvantage as independent predictors of antisocial behavior: A longitudinal adoption study
Source: PLoS One. 2024 Apr 29;19(4):e0301765. doi: 10.1371/journal.pone.0301765 (PMC11057761; doi:10.1371/journal.pone.0301765)
Supplement: S7 Table — (DOCX) [file pone.0301765.s007.docx]

Table S7. Correlations Between Parent Reported ASB Intercept, Slope, and Biological Parent SES in Nonadoptees

|  | Intercept | | Slope | |
| --- | --- | --- | --- | --- |
| *N ­=* 491 | Biological Parent SES | | | |
|  | *r* | *p* | *r* | *p* |
| Girls | .00 [-.22, .21] | .97 | -.27* [-.51, -.04] | .02 |
| Boys | -.03 [-.22, .16] | .76 | -.13 [-.38, .11] | .29 |
| *N =* 507 | ND | | | |
|  | *r* | *p* | *r* | *p* |
| Girls | .14 [-.09, .38] | .23 | -.08 [-.39, .22] | .60 |
| Boys | .04 [-.16, .24] | .71 | .05 [-.19, .29] | .66 |

**p <*.05

*Note:* Standardized correlations reported.
