# Supplementary material for: An examination of early socioeconomic status and neighborhood disadvantage as independent predictors of antisocial behavior: A longitudinal adoption study
Source: PLoS One. 2024 Apr 29;19(4):e0301765. doi: 10.1371/journal.pone.0301765 (PMC11057761; doi:10.1371/journal.pone.0301765)
Supplement: S8 Table — (DOCX) [file pone.0301765.s008.docx]

Table S8. ASB Hierarchical Factor Regressed on Adoptive and Biological Parent SES and ND in Adoptees: Individuals with ND Data Only

| *N =* 211 | Adoptive Parent SES | | | ND | | |
| --- | --- | --- | --- | --- | --- | --- |
|  | β [CI] | SE | *p* | β [CI] | SE | *p* |
| Girls | .01 [-.26, .27] | .14 | .97 | -.06 [-.34, .22] | .14 | .66 |
| Boys | -.18 [-.44, .07] | .13 | .21 | .16 [-.09, .41] | .13 | .21 |
| *N =* 211 | Biological Parent SES | | | ND | | |
|  | β [CI] | SE | *p* | β [CI] | SE | *p* |
| Girls | .31* [-.03, .59] | .14 | .03 | -.50 [-.32, .22] | .14 | .71 |
| Boys | -.01 [-.29, .28] | .15 | .97 | .15 [-.11, .40] | .13 | .25 |

*Note:* β = standardized regression coefficient; “CI” = confidence interval; “SE” = standard error

Model fit for model examining adoptive parent SES: χ^2^(950) = 1068.60, *p* = 0.004, RMSEA = .03, CFI = .98

Model fit for model examining biological parent SES of adoptees: χ^2^(440) = 502.25, *p* = 0.02, RMSEA = .04, CFI = .98
