# Supplementary material for: An examination of early socioeconomic status and neighborhood disadvantage as independent predictors of antisocial behavior: A longitudinal adoption study
Source: PLoS One. 2024 Apr 29;19(4):e0301765. doi: 10.1371/journal.pone.0301765 (PMC11057761; doi:10.1371/journal.pone.0301765)
Supplement: S9 Table — (DOCX) [file pone.0301765.s009.docx]

Table S9. ASB Hierarchical Factor Regressed on Biological Parent SES and ND in Nonadoptees: Individuals with ND Data Only (*N* = 365)

|  | Biological Parent SES | | | ND | | |
| --- | --- | --- | --- | --- | --- | --- |
|  | β [CI] | SE | *p* | β [CI] | SE | *p* |
| Girls | -.06 [-.32, .19] | .13 | .63 | .07 [-.16, .30] | .12 | .57 |
| Boys | -.29** [-.50, -.09] | .10 | .004 | .05 [-.18, .28] | .12 | .66 |

**non-FDR corrected *p <* .01

*Note:* β = standardized regression coefficient; “CI” = confidence interval; “SE” = standard error

Model fit for model examining biological parent SES of nonadoptees: χ^2^(950) = 1068.60, *p* = 0.004, RMSEA = .03, CFI = .9
