# Supplementary material for: An examination of early socioeconomic status and neighborhood disadvantage as independent predictors of antisocial behavior: A longitudinal adoption study
Source: PLoS One. 2024 Apr 29;19(4):e0301765. doi: 10.1371/journal.pone.0301765 (PMC11057761; doi:10.1371/journal.pone.0301765)
Supplement: S10 Table — (DOCX) [file pone.0301765.s010.docx]

Table S10. Parent Reported ASB Slope, Intercept, and Correlations Between Slope and Intercept: Individuals with ND Data Only (*N =* 428)

|  | Slope | | Intercept | | Slope with Intercept |
| --- | --- | --- | --- | --- | --- |
|  | Mean | Variance | Mean | Variance |  |
| Adoptees |  |  |  |  |  |
| Girls | -0.26 | 1.95 | 0.00 | 1.20* | -0.13 |
| Boys | -0.44* | 1.65 | 0.18 | 1.70 | -0.06 |
| Nonadoptees |  |  |  |  |  |
| Boys | -1.20*** | 1.63 | -0.18 | 0.99 | -0.22 |
| Girls | -0.99*** | 1.53 | 0.17 | 1.41 | -0.31 |

**p <*.05, ***p* < .01, ****p* ≤ .001

*Note:* Standardized means and correlations between slope and intercept reported.
