# Supplementary material for: An examination of early socioeconomic status and neighborhood disadvantage as independent predictors of antisocial behavior: A longitudinal adoption study
Source: PLoS One. 2024 Apr 29;19(4):e0301765. doi: 10.1371/journal.pone.0301765 (PMC11057761; doi:10.1371/journal.pone.0301765)
Supplement: S11 Table — (DOCX) [file pone.0301765.s011.docx]

Table S11. Parent Reported ASB Intercept Regressed on Biological and Adoptive Parent SES and ND in Adoptees: Individuals with ND Data Only

| *N =* 211 | Adoptive Parent SES | | | ND | | |
| --- | --- | --- | --- | --- | --- | --- |
|  | β [CI] | SE | *p* | β [CI] | SE | *p* |
| Girls | -.24 [-.49, .02] | .13 | .07 | .05 [-.19, .29] | .12 | .69 |
| Boys | -.27* [-.54, .00] | .14 | .05 | .05 [-.18, .27] | .12 | .69 |
| *N =* 211 | Biological Parent SES | | | ND | | |
|  | β [CI] | SE | *p* | β [CI] | SE | *p* |
| Girls | .20 [-.07, .48] | .14 | .15 | -.02 [-.26, .22] | .12 | .87 |
| Boys | .07 [-.17, .32] | .13 | .55 | .11 [-.11, .33] | .11 | .34 |

*non-FDR corrected *p <* .05

*Note:* β = standardized regression coefficient; “CI” = confidence interval; “SE” = standard error

Model fit for model examining adoptive parent SES: χ^2^(296) = 337.15*, p =* 0.05; RMSEA = .03, CFI = .99

Model fit for model examining biological parent SES: χ^2^(143) = 168.19*, p =* 0.07, RMSEA = .04, CFI = .99
