# Supplementary material for: An examination of early socioeconomic status and neighborhood disadvantage as independent predictors of antisocial behavior: A longitudinal adoption study
Source: PLoS One. 2024 Apr 29;19(4):e0301765. doi: 10.1371/journal.pone.0301765 (PMC11057761; doi:10.1371/journal.pone.0301765)
Supplement: S12 Table — (DOCX) [file pone.0301765.s012.docx]

Table S12. Parent Reported ASB Slope Regressed on Biological and Adoptive Parent SES and ND in Adoptees: Individuals with ND Data Only

| *N =* 211 | Adoptive Parent SES | | | ND | | |
| --- | --- | --- | --- | --- | --- | --- |
|  | β [CI] | SE | *p* | β [CI] | SE | *p* |
| Girls | .15 [-.11, .40] | .13 | .27 | -.19 [-.45, .07] | .13 | .16 |
| Boys | -.00 [-.35, .35] | .18 | 1.00 | .19 [-.20, .57] | .20 | .34 |
| *N =* 211 | Biological Parent SES | | | ND | | |
|  | β [CI] | SE | *p* | β [CI] | SE | *p* |
| Girls | .27 [-.13, .68] | .21 | .19 | -.20 [-.49, .08] | .15 | .16 |
| Boys | -.29* [-.56, -.02] | .14 | .04 | .13 [-.27, .52] | .20 | .53 |

*non-FDR corrected *p <* .05

*Note:* β = standardized regression coefficient; “CI” = confidence interval; “SE” = standard error

Model fit for model examining adoptive parent SES: χ^2^(296) = 337.15*, p =* 0.05; RMSEA = .03, CFI = .99

Model fit for model examining biological parent SES: χ^2^(143) = 168.19*, p =* 0.07, RMSEA = .04, CFI = .99
