# Supplementary material for: An examination of early socioeconomic status and neighborhood disadvantage as independent predictors of antisocial behavior: A longitudinal adoption study
Source: PLoS One. 2024 Apr 29;19(4):e0301765. doi: 10.1371/journal.pone.0301765 (PMC11057761; doi:10.1371/journal.pone.0301765)
Supplement: S13 Table — (DOCX) [file pone.0301765.s013.docx]

Table S13. Parent Reported ASB Intercept Regressed on Biological Parent SES and ND in Nonadoptees: Individuals with ND Data Only (*N =*365)

|  | Biological Parent SES | | | ND | | |
| --- | --- | --- | --- | --- | --- | --- |
|  | β [CI] | SE | *p* | β [CI] | SE | *p* |
| Girls | .10 [-.19, .39] | .15 | .49 | .18 [-.41, .13] | .12 | .15 |
| Boys | -.10 [-.32, .13] | .12 | .41 | .03 [-.16, .22] | .10 | .78 |

*non-FDR corrected *p <* .05

*Note:* β = standardized regression coefficient; “CI” = confidence interval; “SE” = standard error

Model fit for model examining biological parent SES for nonadoptees: χ^2^(296) = 337.15*, p =* 0.05; RMSEA = .03, CFI = .99
