# Supplementary material for: An examination of early socioeconomic status and neighborhood disadvantage as independent predictors of antisocial behavior: A longitudinal adoption study
Source: PLoS One. 2024 Apr 29;19(4):e0301765. doi: 10.1371/journal.pone.0301765 (PMC11057761; doi:10.1371/journal.pone.0301765)
Supplement: S14 Table — (DOCX) [file pone.0301765.s014.docx]

Table S14. Parent Reported ASB Slope Regressed on Biological Parent SES and ND in Nonadoptees: Individuals with ND Data Only (*N =* 365)

|  | Biological Parent SES | | | ND | | |
| --- | --- | --- | --- | --- | --- | --- |
|  | β [CI] | SE | *p* | β [CI] | SE | *p* |
| Girls | -.25 [-.54, .03] | .15 | .08 | -.14 [-.41, .13] | .14 | .31 |
| Boys | -.18 [-.42, .06] | .12 | .15 | .06 [-.19, .31] | .13 | .65 |

*non-FDR corrected *p <* .05

*Note:* β = standardized regression coefficient; “CI” = confidence interval; “SE” = standard error

Model fit for model examining biological parent SES for nonadoptees χ^2^(296) = 337.15*, p =* 0.05; RMSEA = .03, CFI = .99
