# Supplementary material for: An examination of early socioeconomic status and neighborhood disadvantage as independent predictors of antisocial behavior: A longitudinal adoption study
Source: PLoS One. 2024 Apr 29;19(4):e0301765. doi: 10.1371/journal.pone.0301765 (PMC11057761; doi:10.1371/journal.pone.0301765)
Supplement: Attachment — Submitted filename: Response to Reviewers.docx [file pone.0301765.s015.docx]

February 28, 2024

Bárbara Oliván-Blázquez, Ph.D.

Academic Editor

PLOS ONE

Dear Dr. Oliván-Blázquez,

We thank you and the reviewers for helpful suggestions to our manuscript “An examination of early socioeconomic status and neighborhood disadvantage as independent predictors of antisocial behavior: a longitudinal adoption study.” Please find attached the revision of this manuscript. The portions of the manuscript revised in response to reviewer’s comments are tracked as changes. Below, we have provided responses to the reviewers’ comments in bold font with any revisions in green font.

Sincerely,

Shelley Gresko, M.A.

Doctoral Student in Clinical Psychology

Department of Psychology and Neuroscience

University of Colorado-Boulder

1. When submitting your revision, we need you to address these additional requirements.

Please ensure that your manuscript meets PLOS ONE's style requirements, including those for file naming. The PLOS ONE style templates can be found at

https://journals.plos.org/plosone/s/file?id=wjVg/PLOSOne_formatting_sample_main_body.pdf and

<https://journals.plos.org/plosone/s/file?id=ba62/PLOSOne_formatting_sample_title_authors_affiliations.pdf>.

**We have ensured that our manuscript meets PLOS ONE’s style requirements, including those for file naming.**

2. Note from Emily Chenette, Editor in Chief of PLOS ONE, and Iain Hrynaszkiewicz, Director of Open Research Solutions at PLOS: Did you know that depositing data in a repository is associated with up to a 25% citation advantage (https://doi.org/10.1371/journal.pone.0230416)? If you’ve not already done so, consider depositing your raw data in a repository to ensure your work is read, appreciated and cited by the largest possible audience. You’ll also earn an Accessible Data icon on your published paper if you deposit your data in any participating repository (https://plos.org/open-science/open-data/#accessible-data).

**A portion of our data is currently available through the Harvard Dataverse at** [**https://doi.org/doi:10.7910/DVN/BCDSEU**](https://doi.org/doi:10.7910/DVN/BCDSEU) **(Plomin et al., 2022) and access can be requested by contacting the Manager of Operations for the Henry A. Murray Research Archive, Institute for Quantitative Social Sciences, 1737 Cambridge St, Cambridge, MA 02138, USA., mra@help.hmdc.harvard.edu. Researchers who meet the criteria for access to confidential data may apply for access to data not yet added to the Harvard Dataverse by contacting the corresponding author (shelley.gresko@colorado.edu) and the University of Colorado Boulder Institutional Review Board (**[**irbadmin@colorado.edu)**](mailto:irbadmin@colorado.edu))**.**

3. We note that you have indicated that there are restrictions to data sharing for this study. PLOS only allows data to be available upon request if there are legal or ethical restrictions on sharing data publicly. For more information on unacceptable data access restrictions, please see http://journals.plos.org/plosone/s/data-availability#loc-unacceptable-data-access-restrictions.

If there are ethical or legal restrictions on sharing a de-identified data set, please explain them in detail (e.g., data contain potentially identifying or sensitive patient information, data are owned by a third-party organization, etc.) and who has imposed them (e.g., a Research Ethics Committee or Institutional Review Board, etc.). Please also provide contact information for a data access committee, ethics committee, or other institutional body to which data requests may be sent.

**We have provided the following information on ethical restrictions on sharing a de-identified data set:** **“Given that the data contain potentially identifying and sensitive patient information, data cannot be shared publicly because of restrictions put in place by the University of Colorado Boulder Institutional Review Board. Unidentifiable patient data are in the process of being added to the Harvard Dataverse repository. A portion of this data is currently available through the Harvard Dataverse at** [**https://doi.org/doi:10.7910/DVN/BCDSEU**](https://doi.org/doi:10.7910/DVN/BCDSEU) **(Plomin et al., 2022) and access can be requested by contacting the Manager of Operations for the Henry A. Murray Research Archive, Institute for Quantitative Social Sciences, 1737 Cambridge St, Cambridge, MA 02138, USA., mra@help.hmdc.harvard.edu. Researchers who meet the criteria for access to confidential data may apply for access to data not yet added to the Harvard Dataverse by contacting the corresponding author (shelley.gresko@colorado.edu) and the University of Colorado Boulder Institutional Review Board (**[**irbadmin@colorado.edu)**](mailto:irbadmin@colorado.edu))**.”**

**Plomin, R., DeFries, J. C., & Fulker, D. W. (2022). Colorado Adoption Project, 1976-1989 Version V1) [longitudinal, field study, hereditary]. Harvard Dataverse.** [**https://doi.org/doi:10.7910/DVN/BCDSEU**](https://doi.org/doi:10.7910/DVN/BCDSEU)

We will update your Data Availability statement on your behalf to reflect the information you provide.

4. Please include your full ethics statement in the ‘Methods’ section of your manuscript file. In your statement, please include the full name of the IRB or ethics committee who approved or waived your study, as well as whether or not you obtained informed written or verbal consent. If consent was waived for your study, please include this information in your statement as well.

**In our Methods section on page 9, lines 187-191, we include the following: “This project is approved by the University of Colorado Boulder Office of Research Integrity’s Institutional Review Board (protocol number 14-0421). Parents completed written consent and children aged seven and older provided written or verbal assent and children provided written consent at age 16.** **Only participants who provided consent for researchers to geocode first addresses were included in this study.**

5. Please include a copy of Table S16 in Supporting Information which you refer to in your text on page 23.

**Thank you for drawing attention to this discrepancy. The reference to Table S16 in Supporting Information was an error and has been deleted.**

5. Review Comments to the Author

Reviewer #1: This study shares important information about the potential impact of socioeconomic status on children's psychological health.

An extensive review of the topic has been carried out as a background, however, reviewing the bibliography used, I consider that it would be necessary to incorporate more current existing bibliography. It is true that it is a project started in 1975, but if the purpose is to give it scope and provide us with evidence of the present, it would be necessary to incorporate more recent information.

**We have added the following studies to include more contemporary references in the introduction section:**

**13. Peverill M, Dirks MA, Narvaja T, Herts KL, Comer JS, McLaughlin KA. Socioeconomic status and child psychopathology in the United States: A meta-analysis of population-based studies. Clin Psychol Rev. 2021 Feb 1;83:101933.**

**14. Chang LY, Wang MY, Tsai PS. Neighborhood disadvantage and physical aggression in children and adolescents: A systematic review and meta-analysis of multilevel studies. Aggress Behav. 2016;42(5):441–54.**

**29. Choi JK, Kelley MS, Wang D. Neighborhood Characteristics, Maternal Parenting, and Health and Development of Children from Socioeconomically Disadvantaged Families. Am J Community Psychol. 2018;62(3–4):476–91.**

**33. Latham RM, Arseneault L, Alexandrescu B, Baldoza S, Carter A, Moffitt TE, et al. Violent experiences and neighbourhoods during adolescence: understanding and mitigating the association with mental health at the transition to adulthood in a longitudinal cohort study. Soc Psychiatry Psychiatr Epidemiol. 2022 Dec 1;57(12):2379–91.**

**41. Burt SA. The Genetic, Environmental, and Cultural Forces Influencing Youth Antisocial Behavior Are Tightly Intertwined. Annu Rev Clin Psychol. 2022;18(1):155–78.**

On the other hand, in Line 46, if "" is part of the objective of the study, I do not consider it so relevant to put this information at the beginning of the introduction without having given justifying arguments.

**We have removed the following sentence “The present study aims to examine whether early childhood socioeconomic status measured in infancy and neighborhood disadvantage independently contribute to level and change of ASB and whether these influences are best explained by gene­–environment correlation or environmental mediation, using a longitudinal adoption design.” in the first paragraph of the introduction per the reviewer’s suggestion.**

Methodologically, it is a study that uses very complex statistical analyses. However, I have doubts about this methodology:

--There is a lack of a contribution of ethical considerations in methodology, which specifies the ethical committee and how the data have been treated, which in this study precisely involving minors has to be very well argued.

**On page 9, lines 187-189, we clarify the following related to ethical considerations: “This project is approved by the University of Colorado Boulder Office of Research Integrity’s Institutional Review Board (protocol number 14-0421). Parents completed written consent and children aged seven and older provided written or verbal assent and children provided written consent at age 16.”**

-Line 160 indicates that they come from data from a longitudinal study. started in 1975 and recruited as indicated on line 171 until 1987.

Being a longitudinal study, it is not indicated if or when these people were recruited. Were they monitored during the recruitment period?

Reviewing the articles you cite (Plomin and DeFries (55) and Rhea et al. (49). ), they also do not specify when follow-up evaluations are done.

**On pages 8-9, lines 169-177, we clarify when parents and siblings were recruited and have added additional information: Prospective recruitment began on January 1st, 1976, and ended on September 30th, 1987. Biological mothers of adoptees were recruited from two large adoption agencies in Colorado. Their children were placed into their adoptive family homes within one year after birth. Social workers matched adoptees with adoptive families on non-proximity of location and similarity of height between adoptive and biological parents. No additional explicit selective placement practices were followed. Seventy-five percent of adoptive parents recruited for the study agreed to participate. Matched control families were recruited from local hospitals. Sibling enrollment and recruitment ended with the last longitudinally followed sibling.**

**On page 9, lines 177-180, we specify how often follow up evaluations were completed and have added information about age at assessment: “Biological and adoptive parents were assessed with a comprehensive battery of psychological measures when children were one year old or younger. Adoptees and nonadoptees were assessed approximately annually either through home or lab visits or telephone interviews, from age one year to 16 years.**

On the other hand, line 191 specifies that the data has been accessed in 2021.

I consider that it would be necessary to better explain what date the data presented in this manuscript corresponds to.

**On page 9, lines 193-196, we have clarified that the raw data collected prospectively beginning in 1976 and was accessed by the authors of the manuscript in 2021 and 2023:** **“Data were collected beginning in 1976. The socioeconomic status and antisocial behavior data were accessed by authors on April 27^th^, 2021, and the neighborhood disadvantage data were finalized and accessed on January 20^th^, 2023 (see Methods section for more details on the neighborhood disadvantage variable).”**

In measures, a description of the sample has been made in terms of the socioeconomic variables (From line 202 to 221, including table 1), of the Neighborhood disadvantage variables (Line 242-246). I consider this information provided to be not so relevant in methodology but rather as presentation and description of the sample at the beginning of the results section.

**We have moved the sample description for SES and ND variables into the results section, under the heading “Sample Description” (pages 14-15).**

As in the introduction, an extensive search has been carried out in the discussion, but also outdated in terms of publication dates.

**We have added the following studies to include more contemporary references in the discussion section:**

**13. Peverill M, Dirks MA, Narvaja T, Herts KL, Comer JS, McLaughlin KA. Socioeconomic status and child psychopathology in the United States: A meta-analysis of population-based studies. Clin Psychol Rev. 2021 Feb 1;83:101933.**

**14. Chang LY, Wang MY, Tsai PS. Neighborhood disadvantage and physical aggression in children and adolescents: A systematic review and meta-analysis of multilevel studies. Aggress Behav. 2016;42(5):441–54.**

**41. Burt SA. The Genetic, Environmental, and Cultural Forces Influencing Youth Antisocial Behavior Are Tightly Intertwined. Annu Rev Clin Psychol. 2022;18(1):155–78.**

**85. Burt SA, Klump KL, Gorman-Smith D, Neiderhiser JM. Neighborhood Disadvantage Alters the Origins of Children’s Nonaggressive Conduct Problems. Clin Psychol Sci. 2016 May 1;4(3):511–26.**

Reviewer #2: General Assessment:

The introduction effectively addresses various aspects related to Antisocial Behavior (ASB), focusing on potential environmental predictors such as parental socioeconomic status and neighborhood disadvantage. It explores the associations between socioeconomic status (SES), neighborhood disadvantage (ND), and ASB across different genders and types of antisocial behaviors. Additionally, the introduction highlights the use of adoption studies to differentiate between passive genetic influences (passive rGE) and environmental mediation in understanding ASB. The topic is highly relevant to the field, and the research question is well-defined. The study investigated the influence of early socioeconomic status (SES) and neighborhood disadvantage (ND) on antisocial behavior (ASB) using a longitudinal adoption design. The research, based on data from the Colorado Adoption Project, found that lower SES was a significant predictor of ASB in nonadoptive families, while ND did not show a significant association with ASB. The study did not provide conclusive evidence regarding the genetic versus environmental factors influencing the relationship between SES, ND, and ASB, as associations were not consistently significant across sex or adoptive status. However, in nonadopted individuals, lower SES was consistently associated with an increased risk of ASB, aligning with previous research findings.

First of all, to make sure that the manuscript adheres to the journal's guidelines and formatting requirements, please, check the “Instructions for the authors” document:

https://journals.plos.org/plosone/s/submission-guidelines#loc-financial-disclosure-statement

Apart from that, please consider the below comments to strengthen the study as reported, which reflect my responses to general checklists:

Major Comments:

• Line 182. In terms of racial diversity, could you provide more information on the distribution within the five reported categories OR whether there were any notable differences in outcomes based on race?

**We have included more specific demographic information regarding percentage of participants who belonged to each race on page 9, lines 180-184:**

**“The sample is over 90% White and is demographically representative of the Denver Metropolitan area at the time of recruitment (55). Participants self-reported their race as one of five categories: Alaskan Native/American Indigenous (1.4%), Asian (4.7), Black (0.6%), White (91.6%), and more than one race (0.9%), or unknown or not reported (0.9%).”**

**Given that the number of non-White subjects is very small, we do not have the power to detect any significant differences in outcomes based on race.**

• Line 200. The paragraph mentions that only paternal occupational status was examined due to limited mothers working outside the home at the study's initiation. Could you provide more context on this decision and its potential impact on the comprehensiveness of the SES assessment?

**On page 10, lines 205-209, we have provided additional context regarding the decision to include only paternal occupational status: “Only paternal occupational status was examined****; given that the adoption agencies required that one parent be an at-home parent and given the limited number of mothers working outside of the home when the study was initiated, including occupational ratings for mothers would not have meaningfully added to the assessment of familial SES during infancy.”**

**We have also included this in the limitations section on page 30, lines 492-495: “Second, only paternal occupational status was examined since the adoption agencies required one parent be an at-home parent and few mothers worked outside of the home when the study was initiated, but SES estimates may be low for families with mothers working outside of the home.”**

• Line 259. The decision to conduct confirmatory factor analyses (CFA) for examining aggression, delinquency, and overall Antisocial Behavior (ASB) is mentioned. Could you provide insights into the specific reasons or theoretical justifications for choosing CFA over exploratory factor analysis (EFA) in this study?

**Thank you for the question. Overall, we were interested in testing a specific model rather than seeing how many or which ASB factors we could find within the CBCL****. Specifically, we conducted CFA as opposed to EFA for aggression and delinquency because these are well established characteristics of antisocial behavior that are differentially observed across sex (e.g., higher aggression in males, higher delinquency in females) and have also been confirmed by prior CFA (Fergusson et al., 1994; Greenbaum & Dedrick, 1998). The ASB, or externalizing scale, in the CBCL has also been confirmed by prior CFA (i.e., Greenbaum & Dedrick, 1998).** **We created a hierarchical factor with loadings on each year to capture a broad construct of ASB across childhood and adolescence.**

**We have added the following into the manuscript for clarification on page 12, lines 249-251 and 254-255:**

**“We used CFA instead of exploratory factor analysis (EFA) to capture a broad construct of ASB across childhood and adolescence. The latent hierarchical ASB factor model fit the data well, χ2(806) = 898.55, p = .01, RMSEA = 0.02, CFI = 0.99.**

**We also created separate delinquency and aggression factors comprised of hierarchical latent factors with loadings on parent and teacher reported aggression and delinquency scales. CFA, as opposed to EFA, were conducted for aggression and delinquency because these are well-established characteristics of antisocial behavior (70,71).”**

**Fergusson, D. M., Horwood, L. J., & Lynskey, M. T. (1994). Structure of DSM-III-R Criteria for Disruptive Childhood Behaviors: Confirmatory Factor Models. *Journal of the American Academy of Child & Adolescent Psychiatry*, *33*(8), 1145–1157. https://doi.org/10.1097/00004583-199410000-00010**

**Greenbaum, P. E., & Dedrick, R. F. (1998). Hierarchical confirmatory factor analysis of the Child Behavior Checklist/4–18. *Psychological Assessment*, *10*(2), 149–155. https://doi.org/10.1037/1040-3590.10.2.149**

• Line 476. The mention of potential changes in SES and ND in later childhood or adolescence raises questions about the dynamics of these factors over time. Could you discuss how these potential changes might influence the study's results and interpretations, and suggest ways to address this limitation in future research?

**On page 30, lines 496-501, we have added more discussion regarding how changes in SES or ND may influence the study’s results and interpretations and have suggested ways to address this limitation in future research:**

**“Changes in familial SES or ND may have occurred over the course of the study and our estimates do not capture how those changes or SES and ND in later childhood or adolescence influence ASB, although changes in SES and ND may play a role in ASB change over time. Children may be more likely to engage in delinquent behaviors during years when their parents’ SES is lower (82) and ASB may decrease if children are relocated from high to low ND environments (53,82). Research addressing this issue by assessing familial SES and ND along with ASB prospectively and regularly is needed.”**

Minor Comments:

• In line 260, page 13. There is a symbol that is not displayed well. Same in line 280, 299.

**Thank you for this suggestion. We have ensured that the chi-squared symbol is visible throughout the manuscript.**
